# Cardiac surgery-associated acute kidney injury in neonatal Norwood procedure: incidence, risk factors and impact on mortality and outcomes

**DOI:** 10.1093/icvts/ivaf132

**Published:** 2025-06-03

**Authors:** Laetitia Eberle, Muneaki Matsubara, Jonas Palm, Thibault Schaeffer, Takuya Osawa, Carolin Niedermaier, Paul Philipp Heinisch, Nicole Piber, Gunter Balling, Alfred Hager, Peter Ewert, Jürgen Hörer, Masamichi Ono

**Affiliations:** Department of Congenital and Pediatric Heart Surgery, German Heart Center Munich, Technische Universität München, Munich, Germany; Division of Congenital and Pediatric Heart Surgery, University Hospital of Munich, Ludwig-Maximilians-Universität München, Munich, Germany; Europäisches Kinderherzzentrum München, Munich, Germany; Department of Congenital and Pediatric Heart Surgery, German Heart Center Munich, Technische Universität München, Munich, Germany; Division of Congenital and Pediatric Heart Surgery, University Hospital of Munich, Ludwig-Maximilians-Universität München, Munich, Germany; Europäisches Kinderherzzentrum München, Munich, Germany; Department of Congenital Heart Disease and Pediatric Cardiology, German Heart Center Munich, Technische Universität München, Munich, Germany; Department of Congenital and Pediatric Heart Surgery, German Heart Center Munich, Technische Universität München, Munich, Germany; Division of Congenital and Pediatric Heart Surgery, University Hospital of Munich, Ludwig-Maximilians-Universität München, Munich, Germany; Europäisches Kinderherzzentrum München, Munich, Germany; Department of Congenital and Pediatric Heart Surgery, German Heart Center Munich, Technische Universität München, Munich, Germany; Division of Congenital and Pediatric Heart Surgery, University Hospital of Munich, Ludwig-Maximilians-Universität München, Munich, Germany; Europäisches Kinderherzzentrum München, Munich, Germany; Department of Congenital and Pediatric Heart Surgery, German Heart Center Munich, Technische Universität München, Munich, Germany; Division of Congenital and Pediatric Heart Surgery, University Hospital of Munich, Ludwig-Maximilians-Universität München, Munich, Germany; Europäisches Kinderherzzentrum München, Munich, Germany; Department of Congenital and Pediatric Heart Surgery, German Heart Center Munich, Technische Universität München, Munich, Germany; Division of Congenital and Pediatric Heart Surgery, University Hospital of Munich, Ludwig-Maximilians-Universität München, Munich, Germany; Europäisches Kinderherzzentrum München, Munich, Germany; Department of Cardiovascular Surgery, German Heart Center Munich, Technische Universität München, Munich, Germany; Department of Congenital Heart Disease and Pediatric Cardiology, German Heart Center Munich, Technische Universität München, Munich, Germany; Department of Congenital Heart Disease and Pediatric Cardiology, German Heart Center Munich, Technische Universität München, Munich, Germany; Department of Congenital Heart Disease and Pediatric Cardiology, German Heart Center Munich, Technische Universität München, Munich, Germany; Department of Congenital and Pediatric Heart Surgery, German Heart Center Munich, Technische Universität München, Munich, Germany; Division of Congenital and Pediatric Heart Surgery, University Hospital of Munich, Ludwig-Maximilians-Universität München, Munich, Germany; Europäisches Kinderherzzentrum München, Munich, Germany; Department of Congenital and Pediatric Heart Surgery, German Heart Center Munich, Technische Universität München, Munich, Germany; Division of Congenital and Pediatric Heart Surgery, University Hospital of Munich, Ludwig-Maximilians-Universität München, Munich, Germany; Europäisches Kinderherzzentrum München, Munich, Germany

**Keywords:** acute kidney injury, Norwood procedure, neonates, risk factors

## Abstract

**OBJECTIVES:**

Acute kidney injury commonly complicates congenital heart surgery with cardiopulmonary bypass, increasing morbidity and mortality. This study aimed to evaluate risk factors for postoperative acute kidney injury and its impact on outcomes after the Norwood procedure.

**METHODS:**

Neonates undergoing the Norwood procedure from 2001 to 2022 were reviewed. Using modified neonatal Kidney Disease Improving Global Outcomes criteria, we assessed acute kidney injury and analysed its risk factors and impact on survival.

**RESULTS:**

Among the 355 patients who were included, severe acute kidney injury occurred in 100 (28.2%). Risk factors were low weight at Norwood <2.5 kg (odds ratio: 3.0, *P* = 0.015) and extracorporeal membrane oxygenation support (odds ratio: 2.2, *P* = 0.013). Shunt type was not identified as a risk (*P* = 0.317). Acute kidney injury was an independent risk factor for in-hospital death (odds ratio 2.3, *P* = 0.010) but did not influence survival after hospital discharge (hazard ratio: 1.5, *P* = 0.230). The hazard ratio for mortality in patients with acute kidney injury compared to patients without acute kidney injury was 2.5, *P* < 0.001 with a modified Blalock–Taussig–Thomas shunt and 1.9, *P* = 0.010 with a right ventricle-to-pulmonary artery conduit.

**CONCLUSIONS:**

Severe acute kidney injury occurred in approximately a quarter of patients after the Norwood procedure and is an independent risk for in-hospital mortality, both in patients with a modified Blalock–Taussig–Thomas shunt and right ventricle-to-pulmonary artery conduit.

## GRAPHICAL ABSTRACT

**Figure:**
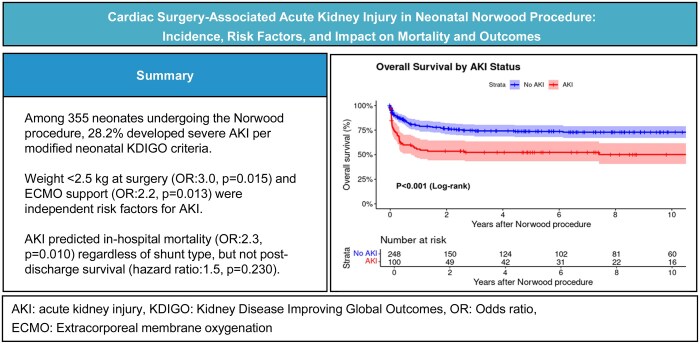

## INTRODUCTION

Acute kidney injury (AKI) is a notable complication following congenital heart surgery, with a reported incidence of 9.6–52% and has been strongly associated with postoperative mortality, especially in young patients [1, 2]. Neonates are at particular risk of postoperative AKI due to their physiological immaturity and unique perinatal factors [3]. The aetiology of AKI is multifactorial, involving complex interactions between the non-physiological circulatory dynamics of cardiopulmonary bypass (CPB), the ischaemia during the aortic cross-clamp, inflammatory responses triggered by blood contact with the extracorporeal circuit and mechanical haemolysis [1–3]. Therefore, AKI is considered a poor prognostic factor leading to prolonged duration of ventilation, increased need for vasoactive drug support, prolonged intensive care unit (ICU) and hospital stay, and even increased mortality [4].

Among congenital heart diseases, hypoplastic left heart syndrome (HLHS) is one of the most complex and difficult to manage [5, 6]. The Norwood procedure, performed as the first stage palliation for HLHS and its variants, is a high-risk operation for neonates, with an elevated risk of postoperative AKI [5–8]. In particular, there is little understanding of the differential relationship between AKI, shunt type and mortality after the Norwood procedure. This relationship is important as shunt type markedly alters postoperative physiology in ways that may affect renal perfusion. However, research focusing on the incidence of AKI following the Norwood procedure, its risk factors and its short- and long-term impacts is limited [7, 8]. The objectives of this study are to determine the incidence of AKI after the Norwood procedure, to evaluate risk factors for the development of AKI, and to assess outcomes in patients who develop AKI.

## METHODS

### Ethical statement

The Institutional Review Board of the Technical University of Munich approved the study (2024–334-S-CB on 08 July 2024). Because of the retrospective nature of the study, the need for individual patient consent was waived. Any collection and storage of data from research participants for multiple and indefinite use is consistent with requirements outlined in the WMA Declaration of Taipei. The ethics committee approved the establishment and monitor the ongoing use of databases.

### Patients and data collection

Medical records of neonates with HLHS and its variants who underwent the Norwood procedure at the German Heart Center Munich from January 2001 to September 2022 were reviewed. Patients who received the Norwood procedure beyond the neonatal period and those who underwent bilateral pulmonary artery banding were excluded. Patient information was obtained from our single-ventricle database, which includes outcomes of subsequent palliative stages, including bidirectional cavopulmonary shunt (BCPS), and total cavopulmonary connection (TCPC). For the monitoring of renal function, blood tests measured creatinine, urea, sodium and potassium levels. Samples were taken before surgery, at admission to the ICU, and every morning for the next week after surgery. The follow-up period for each patient was defined as the interval between the Norwood procedure and the most recent clinical examination. For deceased patients, follow-up was censored at the time of death.

### Operative techniques and ICU management

Operative techniques for the Norwood procedure have been previously reported [9]. Notably, since 2009, cerebral perfusion during aortic clamping has been performed. Up to now, no abdominal perfusion during aortic cross-clamping has been established. Norwood procedures were performed by seven experienced congenital heart surgeons, with four surgeons operating in the first half of the study period (2001–11) and five in the second half (2012–22). The shunt choice of a right ventricle-to-pulmonary artery conduit (RVPAC) or a modified Blalock–Taussig–Thomas shunt (MBTTS) was made by surgeon’s decision. All patients received modified ultrafiltration after waning from CPB. Postoperative fluid management was performed according to our institutional management protocol (Supplementary Table S1). Peritoneal dialysis (PD) was performed at the discretion of the paediatric intensivist. Support of veno-arterial extracorporeal membrane oxygenation (ECMO) was performed through central cannulation of the ascending aorta and the right atrium.

### Diagnosis and treatment of AKI

AKI was defined by the following modified version of the neonatal Kidney Disease Improving Global Outcomes (KDIGO) using measured creatinine levels: stage 1 is an absolute increase in serum creatinine level of 0.3 mg/dl or 1.5 times baseline, stage 2 is two times baseline, and stage 3 is three times baseline, or when dialysis is required. Severe AKI was defined as KDIGO stage 2 or 3 (at least a 2-fold increase in serum creatinine from baseline) [10, 11]. AKI was assessed based on serum creatinine levels recorded preoperatively and on the first to third postoperative days (PODs). Baseline serum creatinine was defined as the lowest value before cardiac surgery. Serum creatinine change (ΔsCre) was expressed as the percentage change in serum creatinine levels compared to the baseline values.

### Management of AKI

Since AKI has a wide variety of causes, our policy is to diagnose AKI at an early stage and appropriately manage it as follows: (1) maintain adequate intravascular volume (infusion therapy), (2) adjust renal perfusion pressure (appropriate use of inotropic agents and vasoconstrictors/dilators) and (3) promptly treat electrolyte abnormalities, acid-base imbalance, fluid overflow and uraemia resulting from renal impairment. However, if patients do not respond to the above-mentioned medical therapies, renal replacement therapy (RRT), such as PD or continuous RRT (especially during ECMO management), is used in combination. Drug non-response was defined as persistent oliguria (<0.5 ml/kg/h) and/or worsening fluid overload despite optimal diuretic therapy (furosemide >2 mg/kg/day) for >24 h according to our institutional protocol. The initiation of RRT was based on the following criteria: severe fluid overload, persistent metabolic acidosis (pH <7.2), hyperkalemia (K+ >6.0 mEq/l) or oliguria (<0.5 ml/kg/h for >12 h) despite optimal medical management. The choice between PD and CRRT was primarily determined by the presence of ECMO support, with CRRT being the preferred modality during ECMO.

### Statistical analysis

Categorical variables are shown as numbers and percentages, and continuous variables as medians with interquartile ranges (IQRs). Levene’s test was used to differentiate between normal and non-normal distributions. A chi-squared test was used for categorical data analysis. Independent-sample t-tests were utilized for normally distributed continuous variables, whereas Mann–Whitney U-tests were used for non-normally distributed variables. A two-way repeated-measures analysis of variance was performed to test changes in serum creatinine over time and changes between survivors and non-survivors, with time as a repeated measure. Both logistic and Cox proportional hazards regression analyses were performed. For all analyses, potential risk factors were first evaluated using univariable analysis, and variables with *P* < 0.1 were included in the initial multivariable models. Variables with *P* < 0.05 in multivariable analysis were retained in the final models. For binary outcomes, including AKI and hospital mortality, odds ratios (ORs) with 95% confidence intervals (CIs) were calculated using multivariable logistic regression. For survival analysis, death was treated as the event of interest, while patients were censored at the time of transplantation or last follow-up. Survival probabilities were estimated using the Kaplan–Meier curve, and survival curves were compared using the log-rank test. The median follow-up time was calculated using the inverse Kaplan–Meier method, where death was treated as a censoring event and last follow-up or transplantation as events. Statistical analyses were performed using Windows SPSS version 28.0 (IBM, Ehningen, Germany) and R-statistical software (R Foundation for Statistical Computing, Vienna, Austria).

## RESULTS

### Patient characteristics

Table 1 shows the AKI distributions of 355 neonates. Of those who developed AKI, 95 (26.8%) were classified as at risk (stage 1), 51 (14.4%) as injury (stage 2) and 49 (13.8%) as failure (stage 3). Severe AKI, determined by the combination of stages 2 and 3, was observed in 100 (28.2%) patients. Patient characteristics with and without severe AKI are shown in Table 2. There was no difference in primary diagnosis or associated anomalies between patients with and without severe AKI. Era analysis was performed between the early (2001–11) and the late era (2012–22), with no significant difference in the incidence of severe AKI. The detailed comparison is shown in Supplementary Table S2. Perioperative variables and mortality data are presented in Table 3. Patients with severe AKI were more likely to weigh less than 2.5 kg at the time of the Norwood procedure than those without severe AKI (12.0% vs 3.9%, *P* = 0.005). Patients with severe AKI had longer CPB times of >150 minutes than those without severe AKI (44.0% vs 32.5%, *P* = 0.043). Postoperatively, patients with severe AKI had a longer ventilator stay (7 vs 5 days, *P* = 0.039) and a longer median ICU stay (17 vs 12 days, *P* < 0.036) than those without severe AKI. ECMO support was more frequently needed in the severe AKI group than those without severe AKI (24.0% vs 11.0%, *P* = 0.002). In-hospital mortality was higher in patients with severe AKI than in those without severe AKI (34.0% vs 14.9%, *P* < 0.001).

**Table 1:** Acute kidney injury distributions

Modified neonatal KDIGO	None	Risk (Stage 1)	Injury (Stage 2)	Failure (Stage 3)

AKI definition	No severe AKI		Severe AKI	
Number (%)	160 (45.0)	95 (26.8)	51 (14.4)	49 (13.8)

**Table 2:** Baseline cohort characteristics

Variables: *N* (%) or median (IQR)	Total	Severe AKI	No severe AKI	*P*-value
Number of patients	355	100 (28.2)	255 (71.8)	
Male sex	243 (68.5)	66 (66.0)	177 (69.4)	0.534
Premature birth	53 (14.9)	15 (15.0)	38 (14.9)	0.905
Gestational age (week)	39 (38–40)	39 (38–40)	39 (38–40)	0.985
Birth weight <2.5 kg	26 (7.3)	11 (11.0)	15 (5.9)	0.095
Genetic anomaly	17 (4.8)	3 (3.0)	14 (5.4)	0.320
Extracardiac anomaly	47 (13.2)	14 (14.0)	33 (12.9)	0.801
Primary diagnosis				
HLHS	289 (81.4)	82 (82.0)	207 (81.2)	0.858
Tricuspid atresia	17 (4.8)	3 (3.0)	14 (5.5)	0.323
DILV	28 (7.9)	12 (12.0)	16 (6.3)	0.072
UAVSD	15 (4.2)	4 (4.0)	11 (4.3)	0.895
Associated anomaly				
TGA	42 (11.8)	13 (13.0)	29 (11.4)	0.669
DORV	22 (6.2)	8 (8.0)	14 (5.5)	0.378
CoA	95 (26.8)	28 (28.0)	67 (26.3)	0.741
Dextrocardia	4 (1.1)	1 (1.0)	3 (1.2)	0.887
Heterotaxy	1 (0.3)	0 (0.0)	1 (0.4)	0.531
TAPVC	10 (2.8)	3 (3.0)	7 (2.7)	0.896
Dominant right ventricle	306 (86.2)	85 (85.0)	221 (86.7)	0.682

**Table 3:** Perioperative variables and mortality

Variables: *N*(%) or median (IQR)	Total	Severe AKI	No severe AKI	*P*-value
Number of patients	355	100	255	
Age at Norwood (days)	8 (7–12)	8 (6–11)	8 (7–12)	0.410
Weight at Norwood (kg)	3.2 (2.9–3.5)	3.1 (2.9–3.5)	3.2 (2.9–3.5)	0.341
Weight at Norwood <2.5 kg	22 (6.2)	12 (12.0)	10 (3.9)	**0.005**
Operative data				
CPB time (min)	138 (108–165)	144 (109–166)	136 (108–164)	0.256
CPB time >150 min	127 (35.8)	44 (44.0)	83 (32.5)	**0.043**
AXC time (min)	49 (41–59)	49 (43–61)	49 (40–59)	0.677
Lowest temp. (C)	19 (18–22)	19 (18–22)	19 (18–22)	0.240
Shunt type				
MBTTS	189 (53.2)	49 (49.0)	140 (54.9)	0.316
RVPAC	166 (46.8)	51 (51.0)	115 (45.1)	
Postoperative data				
Peritoneal dialysis	44 (12.4)	44 (44.0)	0 (0.0)	**<0.001**
Cardiac arrest	19 (5.4)	5 (5.0)	14 (5.5)	0.861
ECMO support	52 (14.6)	24 (24.0)	28 (11.0)	**0.002**
Duration of MV (days)	5 (4–9)	7 (5–14)	5 (4–8)	**0.039**
ICU stay (days)	13 (8–21)	17 (11–25)	12 (8–19)	**0.036**
Hospital stay (days)	24 (16–37)	26 (18–47)	24 (15–36)	0.101
Mortality				
Hospital death	73 (20.6)	35 (35.0)	38 (14.9)	**<0.001**
Reached stage II	263 (74.1)	61 (61.0)	202 (79.2)	**<0.001**
Fontan completion	193 (54.4)	44 (44.0)	149 (58.4)	**0.014**

Bold indicates *P* < 0.05.

### Early postoperative renal function

**Figure 1:**
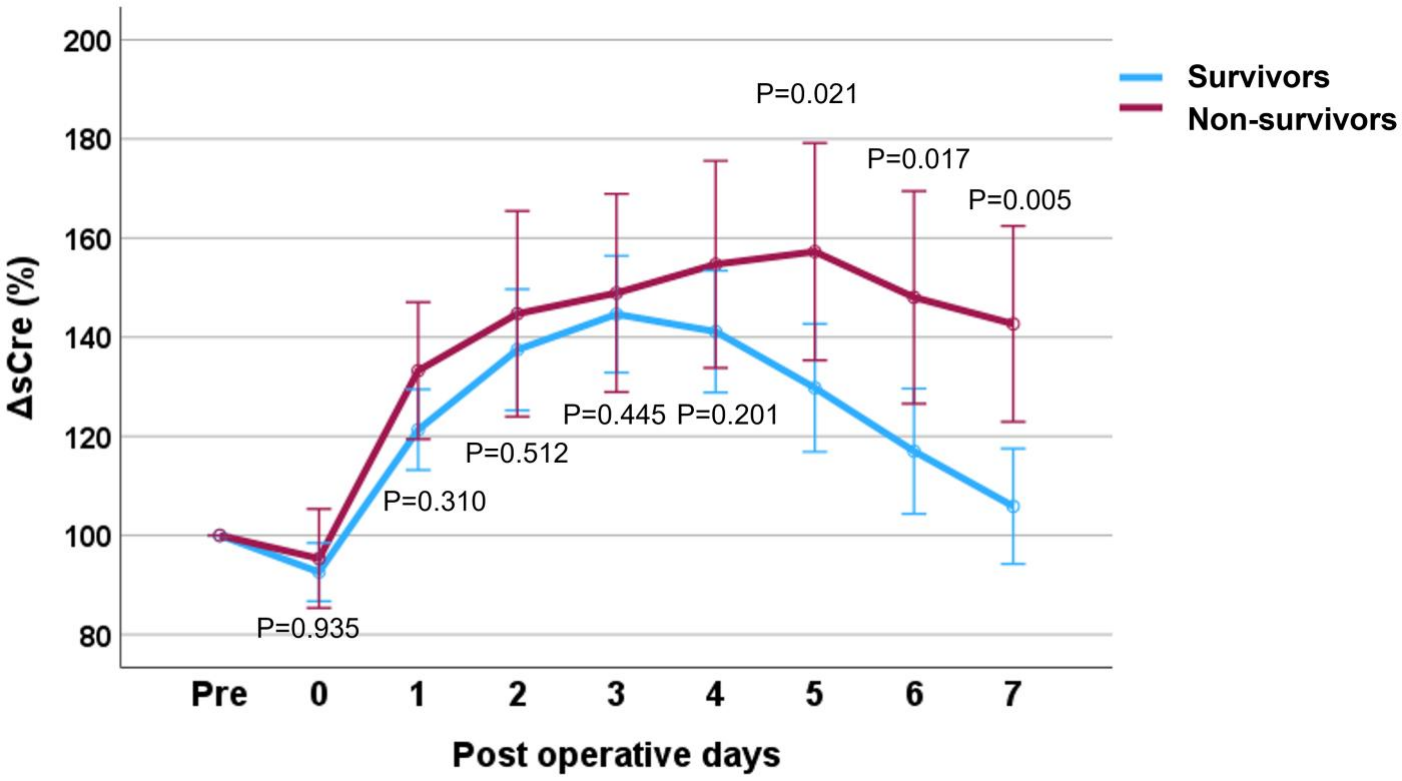
Serum creatinine change in survivors and non-survivors in patients who developed severe AKI after the Norwood procedure. The mean values and standard error of the mean for the % change in serum creatinine from the preoperative value at the nine measurement dates are shown.

Supplementary Table S3 shows the comparison of renal function in patients with severe AKI between survivors and non-survivors. Serum creatinine, sodium and potassium levels were analysed preoperatively and daily from Day 0 to Day 7 postoperatively. Regarding the serum creatinine levels, non-survivors had higher creatinine levels than the survivors after POD 1, and this difference persisted until POD 7 (POD 1, 2, 3, 4, 5, 6, 7; 0.001, 0.008, 0.009, 0.001, 0.001, 0.001 and *P* < 0.001, respectively). Serial ΔsCre in survivors and non-survivors are shown in Fig. 1. Compared to neonates who survived until hospital discharge, neonates who died after surgery had significantly higher ΔsCre from POD 5 to POD 7. In survivors at hospital discharge, the peak serum creatinine levels were reached on POD 3 and then steadily decreased to almost baseline at POD 7. In non-survivors to hospital discharge, serum creatinine peaked on POD 5 and remained high thereafter. Early postoperative serum sodium levels were higher in non-survivors after POD 2, and significant differences were observed until POD 7. There was no difference in serum potassium levels between the survivors and non-survivors at any measured point before and 7 days after the Norwood procedure.

### Haemodialysis for AKI

Table 4 shows the comparison of the outcomes of RRT after the Norwood procedure in survivors and non-survivors at hospital discharge. PD was used in 44 patients (12.4%). The median time from the Norwood procedure to PD was 2.0 days (IQR: 0.0–4.0). The median duration of PDs was 3.5 days (IQR: 2.0–6.3). Non-survivors at hospital discharge were more likely to require PD use than survivors at hospital discharge (32.9% vs 7.1%, *P* < 0.001). Continuous RRT was used in 10 patients (2.8%), 7 in combination with ECMO. Non-survivors were more likely to have RRT than survivors (12.3% vs 0.4%, *P* < 0.001).

**Table 4:** Outcomes of haemodialysis for AKI

Variables: *N* (%) or median (IQR)	Total	Survivors	Non-survivors	*P*-value
Number of patients	355	282 (79.4)	73 (20.6)	
Peritoneal dialysis				
PD	44 (12.4)	20 (7.1)	24 (32.9)	**<0.001**
Time to PD from the Norwood (days)	2.0 (0.0–4.0)	1.0 (0.0–3.0)	3.0 (1.0–6.0)	0.083
Durations of PD (days)	3.5 (2.0–6.3)	3.0 (1.0–5.0)	4.0 (2.0–11.0)	0.066
Renal replacement therapy				
RRT	10 (2.8)	1 (0.4)	9 (12.3)	**<0.001**
RRT+ECMO	7 (2.0)	0 (0.0)	7 (9.6)	**<0.001**

Bold indicates *P* < 0.05.

### Follow-up data

**Figure 2:**
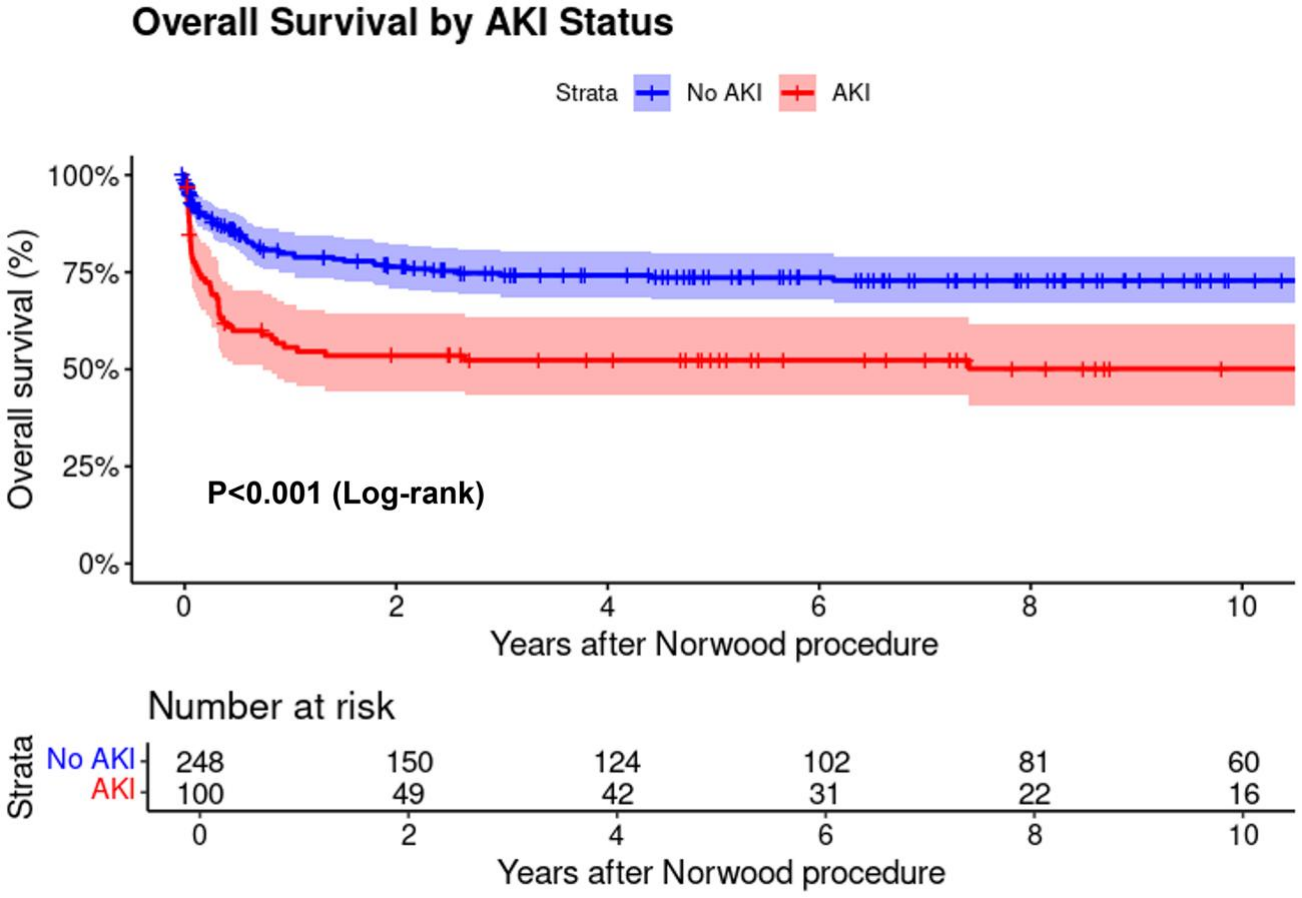
Overall survival after the Norwood procedure by AKI status.

In 282 hospital survivors, the median follow-up was 6.6 years (IQR: 2.4–12.7). The probability of reaching stage 2 (61.0% vs 79.2%, *P* < 0.001) or completion of Fontan surgery (44.0% vs 58.4%, *P* = 0.014) was lower in patients with severe AKI than in those without severe AKI. Overall survival after the Norwood procedure was significantly lower in patients with AKI compared to those without AKI (52.3% vs 73.6% at 5 years, *P* < 0.001, Fig. 2). This survival difference was particularly pronounced in the first year after surgery (55.6% vs 79.8% at 1 year), with both groups showing relatively stable survival rates thereafter.

### Risk factors for severe AKI and in-hospital death

The results of the risk factor analysis are shown in Table 5. Severe AKI was found to be an independent predictor of in-hospital mortality (OR = 2.3, *P* = 0.010). The hazard ratio for mortality in patients with AKI compared to patients without AKI was 2.5 (*P* < 0.001) with a MBTTS and 1.9 (*P* = 0.010) with an RVPAC. Multivariable analysis identified two independent risk factors for severe AKI after the Norwood procedure: weight <2.5 kg at surgery (OR = 3.0, *P* = 0.015) and ECMO support (OR = 2.2, *P* = 0.013).

**Table 5:** Risk factors for hospital death and severe AKI

Risk factors for hospital death						
Variables	Univariable			Multivariable		
	Odds ratio	95% CI	*P*-value	Odds ratio	95% CI	*P*-value
Weight at Norwood <2.5 kg	4.4	1.81–10.53	**0.001**	3.6	1.27–10.01	**0.016**
CPB time >150 min	2.0	1.21–3.44	**0.007**			
ECMO support	16.2	8.18–31.99	**<0.001**	14.2	6.91–29.12	**<0.001**
Severe AKI	3.1	1.80–5.26	**<0.001**	2.3	1.23–4.34	**0.010**

Risk factors for severe AKI						
Variables	Univariable			Multivariable		
	Odds ratio	95% CI	*P*-value	Odds ratio	95% CI	*P*-value
Weight at Norwood <2.5 kg	3.3	1.39–8.00	**0.007**	3.0	1.23–7.33	**0.015**
CPB time >150 min	1.6	1.01–2.62	**0.044**			
ECMO support	2.6	1.40–4.68	**0.002**	2.2	1.19–4.15	**0.013**

Bold indicates *P* < 0.05.

## DISCUSSION

### Incidence of AKI after neonatal cardiac surgery

AKI associated with paediatric cardiac surgery is common with an incidence of 14–67%, and at risk for postoperative mortality [1–2, 12–14]. Currently, there are no well-established criteria for neonatal AKI. A low glomerular filtration rate during the first week after birth may not be accompanied by an obvious change in serum creatinine level [4]. The definition of AKI after neonatal cardiac surgery has not been established, and there are many criteria, such as the pRIFLE, AKIN, KDIGO and modified KDIGO criteria. Lu *et al.* [11] demonstrated that the modified KDIGO criteria are the most reliable. Therefore, we adopted the modified KDIGO criteria, which are specifically relevant for evaluating renal function in neonates undergoing the Norwood procedure. In the present cohort, 28% of the patients experienced severe AKI, which is consistent with previous studies [12–14]. However, our results also showed that another 26% of the patients were classified as stage 1 AKI, demonstrating that AKI is a frequent sequela after the Norwood procedure.

### Impact of AKI on outcome after the Norwood procedure

Many studies demonstrated the negative impact of AKI on outcomes after paediatric cardiac surgery [1–2, 12–14]. In line with these previous reports, our results showed higher in-hospital mortality in patients with AKI compared to those without. We could also demonstrate that an effective therapy that returns serum creatinine level quickly to the baseline is associated with better hospital survival. The long-term outcomes of postoperative AKI in infants and children with congenital heart disease remain to be determined. Wong *et al.* [8] demonstrated that severe AKI after stage 1 palliation was an independent risk factor for developing severe AKI at stage 2 and was associated with prolonged duration of mechanical ventilation following stage 3. Our results demonstrated that survival after hospital discharge was comparable between patients with and without AKI.

On the other hand, our study highlights the economic importance of AKI prevention strategies, as the increase in ICU length of stay and ECMO use associated with AKI is likely to lead to substantial increases in healthcare costs. However, we did not investigate the renal function after the hospital discharge. Information on the incidence and associated risks for postoperative AKI in HLHS patients from multicentre studies is necessary to further understand the long-term burden of severe AKI after staged palliation.

### Risk factors for the development of AKI

Li *et al.* [3] demonstrated that CPB time and age were independently associated with AKI. This study’s multivariable analysis revealed two independent risk factors for AKI: low weight at Norwood (<2.5 kg) and ECMO support. These risk factors suggests that low body weight and haemodynamic instability might be strongly associated with the development of postoperative AKI.

### Prevention and treatment of AKI

Prevention of AKI during and after the Norwood procedure is the most important issue. Rajagopal *et al.* [15] reported that simultaneous brain and visceral perfusion during neonatal aortic arch reconstruction reduced the incidence of AKI. Hammel *et al.* [16] further demonstrated that descending aortic cannulation (DAC) significantly decreased the AKI incidence compared to conventional selective cerebral perfusion (SCP) (5% vs 31%). The benefits of DAC were further validated by Kulyabin *et al.*’s [17] comprehensive study, which showed that DAC not only reduced the number of patients requiring RRT but also lowered the risk of AKI (OR: 0.91, CI: 0.84–0.98) without increasing mortality rates (7.1% in DAC group vs 11.4% in SCP group). While peripheral cannulation through femoral or umbilical arteries has been proposed as an alternative approach, DAC offers superior advantages, including significantly lower flow resistance, the ability to maintain stable perfusion for extended periods and flexibility in surgical timing without compromising end-organ protection [16]. These collective findings consistently demonstrate the effectiveness of DAC in preserving renal function during neonatal aortic arch reconstruction. As for the shunt type, this study demonstrated no significant difference in the incidence of AKI after the Norwood procedure between MBTTS and RVPAC. As a perioperative management strategy, it is essential to provide adequate fluid therapy and appropriate catecholamine administration, monitor renal perfusion pressure and identify high-risk patients (low body weight, long duration of CPB) early. The development of standardized protocols that incorporate such a comprehensive approach is expected to minimize the risk of postoperative AKI.

### Neonatal cardiac surgery-associated AKI and long-term renal outcomes

The relationship between cardiac surgery-associated AKI (CS-AKI) and chronic kidney disease (CKD) after neonatal cardiac surgery remains controversial. While Madsen *et al.* [18] demonstrated that CS-AKI increased CKD risk 3.8-fold over 5 years, studies by Greenberg and Huynh found no significant association [19, 20]. However, all three studies reported high rates of CKD and hypertension (17–30%) regardless of CS-AKI status, suggesting the involvement of multiple contributing factors such as CPB and chronic hypoxaemia. Therefore, long-term renal monitoring is essential for all post-cardiac surgery neonates.

### Study limitations

Limitations of this study include its single-centre, retrospective design. A total of seven surgeons operated Norwood procedures during the study periods, which may lead to a bias. Despite finding no significant difference in AKI incidence between shunt types, the heterogeneity in surgical approach and shunt selection could have influenced postoperative organ perfusion patterns. A more standardized approach to surgical technique and shunt selection in future studies would help better isolate the specific impact of these variables on renal outcomes. The inability to assess urine output in the early postoperative period due to fluid shifts was a study limitation. While serum creatinine was used as the primary marker for AKI, we acknowledge its limitations in neonates due to maternal influence and immature muscle mass. We did not measure several important variables including intraoperative and postoperative renal near-infrared spectroscopy, flow through the femoral or umbilical artery catheters, hepatic enzyme concentrations and urinary biomarkers such as neutrophil gelatinase-associated lipocalin and kidney injury molecule-1 that could enable earlier AKI detection. To address these limitations, we plan to conduct a multicentre study and begin collecting urinary biomarkers in our prospective cohort. Additionally, future studies should incorporate muscle mass-independent markers such as cystatin C and focus on long-term outcomes, including renal function, quality of life and late complications in survivors of Norwood procedure with severe AKI.

## CONCLUSION

Postoperative severe AKI was observed in 28% of the patients after the neonatal Norwood procedure and was associated with in-hospital death and lower survival. Body weight less than 2.5 kg at the Norwood procedure and the need for ECMO support are risks for the development of severe AKI. The incidence of AKI was comparable between the patients with an MBTTS and those with an RVPAC.

## Supplementary Material

ivaf132_Supplementary_Data

## Data Availability

The data used for this study should be distributed on reasonable requirement.

## ABBREVIATIONS

AKI: Acute kidney injury
CI: Confidence interval
CKD: Chronic kidney disease
CPB: Cardiopulmonary bypass
CS-AKI: Cardiac surgery-associated acute kidney injury
DAC: Descending aortic cannulation
ECMO: Extracorporeal membrane oxygenation
HLHS: Hypoplastic left heart syndrome
ICU: Intensive care unit
IQR: Interquartile range
KDIGO: Kidney Disease Improving Global Outcomes
MBTTS: Modified Blalock–Taussig–Thomas shunt
OR: Odds ratio
PD: Peritoneal dialysis
POD: Postoperative day
RRT: Renal replacement therapy
RVPAC: Right ventricle-to-pulmonary artery conduit
sCre: Serum creatinine change
SCP: Selective cerebral perfusion
