# Supplementary material for: Cardiac surgery-associated acute kidney injury in neonatal Norwood procedure: incidence, risk factors and impact on mortality and outcomes
Source: Interdiscip Cardiovasc Thorac Surg. 2025 Jun 3;40(6):ivaf132. doi: 10.1093/icvts/ivaf132 (PMC12165726; doi:10.1093/icvts/ivaf132)
Supplement: ivaf132_Supplementary_Data [file ivaf132_supplementary_data.zip › Supplementary Tables 19032025.docx]

**Supplementary Tables**

**Supplementary Table S1:**

| Supplementary Table S1: Postoperative Fluid Management Protocol | | |
| --- | --- | --- |
| **Parameter** | **Patients < 0.5 m² BSA** | **Patients > 0.5 m² BSA** |
| Total Fluid (Operation Day) | 1000 ml/m² | 750 ml/m² |
| **Monitoring and Balance Management** | | |
| Volume Balance (6hr checks) | Blood products, colloids/crystalloids, drain output | |
| Water Balance (6hr checks) | Infusions, medications, oral intake, urine output | |
| Target | Negative water balance (diuresis >1-2 ml/kg/h) | |
| Daily Fluid Increase | 250 ml/m²/day up to max 2000 ml/m² | |
| **Volume Management for Complications** | | |
| Oliguria Treatment | Furosemide (0.5-1 mg/kg) or Ethacrynic acid (0.5 mg/kg) q6h | |
| Volume Options | RBCs for anemia, FFP for coagulation issues, Crystalloids/colloids for normal Hct | |

BSA: body surface area, hr: hours, q6h: every 6 hours, RBC: red blood cell, FFP: fresh frozen plasma, Hct: Hematocrit

| **Supplementary Table S2:** | | | | |
| --- | --- | --- | --- | --- |
| Supplementary Table S2: Comparison between Early Era and Late Era | | | |  |
| Variables: N(%) or median (IQR) | **Era at Norwood operation** | | **p-value** |  |
|  | **2001-2011** | **2012-2022** |  |  |
| Number of patients | 165 | 190 |  |  |
| Male sex | 119 (72.1) | 124 (65.3) | 0.165 |  |
| Age at Norwood (days) | 8 (7-12) | 8 (6-11) | 0.712 |  |
| Weight at Norwood (kg) | 3.2 (2.9-3.5) | 3.2 (2.9-3.5) | 0.978 |  |
| Weight at Norwood <2.5 kg | 15 (9.1) | 11 (5.8) | 0.226 |  |
| **Primary Diagnosis** | | | |  |
| HLHS | 137 (83.0) | 152 (80.0) | 0.464 |  |
| Tricuspid atresia | 10 (6.1) | 7 (3.7) | 0.296 |  |
| DILV | 9 (5.5) | 19 (10.0) | 0.113 |  |
| UAVSD | 7 (4.2) | 8 (4.2) | 0.988 |  |
| **Operative data** | | | |  |
| CPB time (min) | 111 (92-140) | 152 (131-182) | <0.001 |  |
| CPB time >150 min | 26 (15.8) | 101 (53.2) | <0.001 |  |
| AXC time (min) | 45 (39-55) | 52 (42-63) | <0.001 |  |
| Lowest Temp. (C) | 19 (18-20) | 20 (18-23) | <0.001 |  |
| **Shunt type** | | | |  |
| MBTTS | 61 (37.0) | 128 (67.4) | <0.001 |  |
| RVPAC | 104 (63.0) | 62 (32.6) | <0.001 |  |
| **Postoperative data** | | | |  |
| Severe AKI | 44 (26.7) | 56 (29.5) | 0.558 |  |
| Peritoneal dialysis | 11 (6.7) | 33 (17.4) | 0.002 |  |
| Cardiac arrest | 10 (6.1) | 9 (4.7) | 0.695 |  |
| ECMO support | 10 (7.1) | 42 (22.1) | <0.001 |  |
| Duration of MV (days) | 6 (4-9) | 5 (4-9) | 0.604 |  |
| ICU stay (days) | 13 (9-22) | 14 (8-21) | 0.467 |  |
| Hospital stay (days) | 25 (15-37) | 24 (16-39) | 0.050 |  |
| **Mortality** | | | |  |
| Hospital death | 24 (14.5) | 49 (25.8) | 0.009 |  |
| Reached Stage II | 137 (83.0) | 126 (66.3) | <0.001 |  |
| Fontan completion | 111 (67.3) | 82 (43.2) | <0.001 |  |

**Supplementary Table S3:**

| Supplementary Table S3: Early postoperative data on renal function | | | | |
| --- | --- | --- | --- | --- |
| **Variables: N(%) or median (IQR)** | **Total** | **Survivors** | **Non-survivors** | **p-value** |
| Number of patients | 355 | 282 (79.4) | 73 (20.6) |  |
| **Serum creatinine (mg/dL)** | | | | |
| Preoperative | 0.60 (0.50-0.77) | 0.60 (0.49-0.75) | 0.63 (0.50-0.82) | 0.127 |
| POD 0 | 0.50 (0.41-0.63) | 0.50 (0.40-0.60) | 0.52 (0.45-0.69) | 0.068 |
| POD 1 | 0.70 (0.60-0.80) | 0.68 (0.56-0.80) | 0.80 (0.64-0.90) | **<0.001** |
| POD 2 | 0.78 (0.60-0.95) | 0.73 (0.59-0.90) | 0.88 (0.69-1.09) | **0.008** |
| POD 3 | 0.80 (0.60-1.00) | 0.75 (0.60-1.00) | 0.93 (0.68-1.10) | **0.009** |
| POD 4 | 0.80 (0.58-1.00) | 0.76 (0.56-0.97) | 0.90 (0.70-1.16) | **0.001** |
| POD 5 | 0.70 (0.50-0.96) | 0.64 (0.47-0.90) | 0.88 (0.66-1.25) | **<0.001** |
| POD 6 | 0.60 (0.45-0.90) | 0.60 (0.43-0.80) | 0.80 (0.58-1.18) | **0.001** |
| POD 7 | 0.59 (0.42-0.80) | 0.53 (0.40-0.70) | 0.80 (0.50-1.16) | **<0.001** |
| **ΔsCre (%)** | | | | |
| POD 0 | 88 (73-105) | 87 (73-105) | 88 (71-106) | 0.935 |
| POD 1 | 117 (97-140) | 117 (95-138) | 120 (100-148) | 0.310 |
| POD 2 | 126 (100-154) | 126 (100-150) | 133 (100-167) | 0.512 |
| POD 3 | 131 (104-163) | 131 (106-160) | 143 (104-170) | 0.445 |
| POD 4 | 129 (100-167) | 125 (100-160) | 148 (96-182) | 0.201 |
| POD 5 | 114 (86-155) | 111 (84-143) | 140 (88-186) | **0.021** |
| POD 6 | 102 (75-144) | 100 (75-133) | 137 (81-190) | **0.017** |
| POD 7 | 100 (72-142) | 94 (71-124) | 143 (72-180) | **0.005** |
| **Serum sodium (mEq/L)** | | | | |
| Preoperative | 136 (133-138) | 136 (133-138) | 136 (134-139) | 0.059 |
| POD 0 | 143 (140-146) | 143 (140-146) | 144 (141-148) | 0.051 |
| POD 1 | 146 (143-149) | 146 (143-149) | 146 (143-150) | 0.736 |
| POD 2 | 146 (143-149) | 146 (143-148) | 147 (144-150) | 0.084 |
| POD 3 | 145 (142-148) | 145 (142-148) | 146 (143-149) | **0.045** |
| POD 4 | 144 (140-147) | 143 (140-147) | 146 (141-149) | 0.070 |
| POD 5 | 141 (138-145) | 141 (138-144) | 144 (140-148) | **0.001** |
| POD 6 | 140 (137-144) | 139 (137-142) | 143 (138-148) | **0.002** |
| POD 7 | 139 (136-143) | 139 (136-141) | 143 (137-148) | **<0.001** |
| **Serum potassium (mmol/L)** | | | | |
| Preoperative | 4.25 (3.87-4.65) | 4.24 (3.85-4.64) | 4.32 (3.95-4.77) | 0.157 |
| POD 0 | 3.95 (3.60-4.41) | 3.94 (3.55-4.39) | 4.02 (3.75-4.54) | 0.147 |
| POD 1 | 4.78 (4.29-5.21) | 4.78 (4.29-5.20) | 4.74 (4.27-5.26) | 0.775 |
| POD 2 | 4.19 (3.89-4.56) | 4.13 (3.85-4.49) | 4.41 (4.03-4.71) | 0.053 |
| POD 3 | 4.25 (3.91-4.64) | 4.19 (3.87-4.63) | 4.34 (3.99-4.67) | 0.163 |
| POD 4 | 4.24 (3.98-4.59) | 4.21 (3.91-4.52) | 4.34 (4.04-4.90) | 0.141 |
| POD 5 | 4.27 (3.95-4.63) | 4.23 (3.94-4.58) | 4.35 (3.94-4.64) | 0.332 |
| POD 6 | 4.29 (3.95-4.70) | 4.29 (3.97-4.64) | 4.37 (3.86-4.80) | 0.813 |
| POD 7 | 4.40 (4.08-4.71) | 4.41 (4.07-4.71) | 4.36 (4.12-4.83) | 0.925 |
